# Characterization of a long-term mouse primary liver 3D tissue model recapitulating innate-immune responses and drug-induced liver toxicity

**DOI:** 10.1371/journal.pone.0235745

**Published:** 2020-07-09

**Authors:** Ramona Nudischer, Kasper Renggli, Andreas Hierlemann, Adrian B. Roth, Cristina Bertinetti-Lapatki

**Affiliations:** 1 Roche Pharmaceutical Research and Early Development, Pharmaceutical Sciences, Roche Innovation Center Basel, F. Hoffmann-La Roche Ltd., Basel, Switzerland; 2 Bioengineering Laboratory, Department of Biosystems Science and Engineering, ETH Zürich, Zurich, Switzerland; University of Pittsburgh, UNITED STATES

## Abstract

Three-dimensional liver *in vitro* systems have recently attracted a lot of attention in drug development. These systems help to gain unprecedented insights into drug-induced liver injury (DILI), as they more closely reproduce liver biology, and as drug effects can be studied in isolated and controllable microenvironments. Many groups established human-based *in vitro* models but so far neglected the animal equivalent, although the availability of both models would be desirable. Animal *in vitro* models enable back- and forward translation of *in vitro* and *in vivo* findings, bridge the gap between rodent *in vivo* and human *in vitro* scenarios, and ultimately support the interpretation of data generated with preclinical species and humans. Since mice are often used in drug development and physiologically relevant *in vitro* systems are lacking, we established, for the first time, a mouse liver model that encompasses primary parenchymal and non-parenchymal cells with preserved viability and functionality over three weeks. Using our three-dimensional liver spheroids, we were able to predict the toxicity of known DILI compounds, demonstrated the interaction cascades between the different cell types and showed evidence of drug-induced steatosis and cholestasis. In summary, our mouse liver spheroids represent a valuable *in vitro* model that can be applied to study DILI findings, reported from mouse studies, and offers the potential to detect immune-mediated drug-induced liver toxicity.

## Introduction

In drug development, *in vivo* and *in vitro* experimental model systems are critical components to support the selection of the most promising drug candidate that show high efficacy and raise little safety concerns in order to be approved for clinical trials. The use of rodent *in vivo* studies to investigate off-target effects is key to assess the systemic impact of a compound in a living organism [1]. Safety-related findings in these animal studies are investigated further to identify specific pathways and mechanisms and, most importantly, their potential relevance to humans. For such organ-specific toxicological studies, *in vitro* systems have demonstrated to constitute valuable models for testing compounds due to several reasons: (i) tests are conducted in a defined and controllable microenvironment to assess direct drug effects on organ-specific cells; (ii) *in vitro* tests are more cost- and time-efficient in comparison to *in vivo* studies, and (iii) they require no or little use of animals to support the generation of a preclinical safety package, which is a compilation of data presented to authorities to enable entry into human [2, 3]. This reduction and avoidance of animal use in drug testing is in concordance with the 3R principles, which are aimed at reducing, replacing and refining animal studies.

The liver is one of the major organs that is investigated in preclinical studies, since it is the main organ involved in metabolism and detoxification of drugs. Drug-induced liver injury (DILI) is accountable for high attrition rates in preclinical and clinical studies as well as after market introduction as a consequence of undesired hepatotoxicity [4]. Currently, monolayers of primary hepatocytes are prevailingly used to screen for drug-induced hepatotoxicity or to study the underlying mechanisms [5]. However, it has been shown that primary isolated hepatocytes, kept under 2D culture conditions, rapidly loose viability, phenotype and liver-specific functions [6, 7]. Therefore, many research groups have been working on the development of more complex, physiologically-relevant, three-dimensional (3D) cell-culture systems and have demonstrated their long-term viability, metabolic activity and immune-specific functionality [8–11]. Most groups, however, have focused on human-cell-based 3D systems [12, 13], thereby overlooking the benefits of having a rodent *in vitro* equivalent to compare data between the different species. To de-risk potential safety liabilities during pre-clinical drug development phases, comparing the drug toxicity profile on both *in vitro* models (rodent vs human) is key to understand the human relevance of a particular rodent *in vivo* finding. Ultimately, at this early stages of drug development, this data contributes to the overall safety assessment package of the molecules, supporting decision making on whether to either modify the drug design or even to halt a program, aiming to reduce the probability of drug attrition in the subsequent clinical phases. Although human *in vitro* systems are finally used to support the drug development process in terms of human relevance, rodent systems can help to better understand and translate the effects seen in different species during testing.

Several rat derived 3D liver models for common hepatotoxicity testing have been published [14–19]. However, mice are equally important and commonly used for disease modeling, pharmacology-, efficacy- and safety studies and are often preferred due to their availability, smaller body size, lower costs, ease of handling, and fast reproduction [20]. Furthermore, in the case of biologics, mouse surrogate molecules which have been used to generate a large collection of *in vivo* efficacy data are often available. Recapitulating toxicities detected in mouse *in vivo* studies in a mouse *in vitro* system increases the confidence of the *in vitro* back translation approaches when trying to recapitulate human clinical toxicities to conduct mechanistic investigations later in development.

In order to satisfy the need of a suitable mouse *in vitro* model, we have developed and characterized 3D spheroids from murine primary hepatocytes, which can be cultured in chemically defined, low-serum media. Primary hepatocytes are considered the gold standard and are preferred over cell lines such as HepaRG and HepG2, as the phenotype and functionality of these cell lines differ substantially from those of primary hepatocytes [21, 22]. Additionally, it has been shown that primary hepatocytes, cultured as 3D spheroids, remain stable for a long period of time while retaining *in vivo* characteristics [14, 18, 23–25]. Compared to conventional primary hepatocyte 2D cultures, 3D spheroids do neither dedifferentiate nor lose their metabolic activity [12, 16]. Furthermore, 3D spheroids have shown to form bile canaliculi and to continuously produce albumin and urea, important features, which are not present or decrease rapidly in traditional 2D culture settings [12,15]. In addition, the use of primary liver spheroids is in concordance with the 3R principle, as the use of animals can be massively reduced. An extensive amount of parenchymal and non-parenchymal cells (NPCs) can be isolated from a single mouse liver [26], which can be used to produce thousands of spheroids, generating a large volume of *in vitro* data, where multiple compounds, concentrations, time points, and endpoints can be tested.

This paper presents, for the first time, the establishment of murine liver spheroids and their characterization in terms of long-term viability and functionality. In addition to monoculture spheroids, we also established coculture spheroids, which, in addition to hepatocytes, contain primary and functionally-active Kupffer cells (KCs), stellate cells (SCs), and liver sinusoidal endothelial cells (LSECs) to address immune-related and other, more complex scientific questions. We characterized the model under physiological conditions and demonstrated its application in long-term liver toxicity studies, drug-induced immune-cell-activation assessment and liver-disease modeling, including steatosis and cholestasis. The obtained results suggest that our murine liver 3D spheroids represent a valuable tool to achieve an *in vitro* representation of *in vivo* drug-induced liver-toxicity effects and drug-induced liver diseases.

## Materials and methods

### Mouse liver perfusion and isolation of parenchymal and non-parenchymal cells

Permission for animal studies was obtained from the local regulatory agencies (BS Kantonales Veterinäramt BS, approval number 2838), and all study protocols were in compliance with federal and cantonal guidelines. Mouse liver cells were isolated from 6-18-week-old male C57BL/6 mice (obtained from Charles River, UK) using a two-step collagenase perfusion method as described earlier [27, 28]. Mice were anaesthetized with sodium pentobarbital (120 mg/kg, i.p.). For cell isolation, the perfused liver was excised, and the liver capsule was mechanically disrupted to release the cells into Williams Medium E (WME) (Gibco, Thermo Fisher Scientific, USA). The cell suspension was filtered through a 40 μm cell strainer and centrifuged at 50 g for 3 min at room temperature (RT). The supernatant containing the non-parenchymal-cell (NPC) fraction was transferred into a new tube and stored at 4°C until further processing. The hepatocyte-cell pellet was resuspended in 25 ml WME medium and mixed with 25ml 90% Percoll RTM pH 8.5–9.5 (Sigma-Aldrich, Switzerland). Cells were centrifuged for 10 min at 50 g, and the supernatant containing dead cells was removed. Cells were washed twice with WME (3 min at 50 g) and resuspended in 3D-plating medium (WME, Thawing/Plating Supplement Pack (Thermo Fisher Scientific, USA), 10% Fetal bovine serum (FBS; Gibco, Thermo Fisher Scientific, USA)). For NPC purification, the cell suspension was centrifuged twice at 50 g for 3 minutes to remove remaining hepatocytes. NPCs were collected in the pellet after a final spin at 360 g for 8 minutes. The cell pellet was resuspended in 1x Red Blood Lysis Buffer (BioLegend, USA) and incubated for 3 min on ice. To stop the reaction, WME was added, and the cells were centrifuged for 5 min at 360 g. The cell pellet containing the NPC fraction was washed twice in WME medium and finally resuspended in 3D plating medium. Hepatocyte and NPC count and viability were assessed by trypan blue exclusion and ranged between 85% and 95%. For each study, a minimum of 3 individual experiments were conducted from different mice liver perfusions.

### Spheroid formation and cultivation

For the formation of monoculture spheroids, 1250 hepatocytes were seeded into each well of a 96-well ultra-low attachment plate (PerkinElmer, Germany) and centrifuged for 3 min at 50 g. For coculture spheroids, 1250 hepatocytes and 625 NPCs were seeded into each well. To avoid increased evaporation, a humidified lid (Labcyte, USA), filled with PBS, was placed on each plate. The liver cells self-aggregated and formed compact spheroids within 4–7 days. From day 4 on, 50% of the medium was replaced daily with serum-free maintenance medium (WME with Cell Maintenance Supplement Pack (Thermo Fisher Scientific, USA)).

### Assessment of viability and functionality

Cell viability was assessed by measuring the adenosine triphosphate (ATP) content of single spheroids using the CellTiter-Glo® 3D Cell Viability Assay (Promega, USA) according to the manufacturer’s instruction. Lactate dehydrogenase (LDH) release from damaged cells into the supernatant was measured by the Cytotoxicity Detection Kit (Roche, Switzerland) according to the manufacturer`s instruction. Supernatant was simultaneously collected to quantify albumin release by using the mouse albumin enzyme-linked immunosorbent assay (ELISA) kit (Bethyl laboratories, USA). Additionally, bright-field images were acquired using the Thermo Fisher Scientific ArrayScan and size of each spheroid was automatically calculated using the Thermo Scientific BioApplication.

The activation potential of Kupffer cells and liver sinusoidal endothelial cells was measured by assessing the lipopolysaccharide (LPS)-induced interleukin 6 (IL-6) release. LPS (10 μM, Sigma-Aldrich, Switzerland) was added, and the supernatant was collected after 48 h. IL-6 concentrations were measured by ELISA (BioLegend, USA). For stellate-cell activation, spheroids were treated with different concentrations of transforming growth factor beta TGFβ (Bio-Techne AG, Switzerland) for 72 hours. Induction of smooth-muscle-α-actin (Acta2) gene expression was measured via quantitative real-time polymerase chain reaction (qRT-PCR), described in the section “gene expression analysis”. For the induction of the major histocompatibility complex class II (MHCII) on LSECs, spheroids were treated with 100 ng ml^-1^ tumor-necrosis factor alpha (TNFα; Sigma-Aldrich, Switzerland) for 48 h. H2-Ab1 gene expression was measured via qRT-PCR.

To investigate the crosstalk between parenchymal and non-parenchymal cells, spheroids were either incubated with 50 μM Dexamethasone (Sigma-Aldrich, Switzerland) or 10 μg/ml LPS alone, or as co-treatment with 24 h LPS following 24 h LPS and Dexamethasone together. Cytochrome P450 (Cyp450), as well as IL-6 and TNFα gene expression was measured by qRT-PCR.

If not otherwise stated, a minimum of three independent experiments were performed with at least 3 technical replicates.

### Immunofluorescence

For immunofluorescence staining, 4–8 spheroids were pooled into 1.5 ml Eppendorf tubes and fixed in 4% Paraformaldehyde (PFA) for 1 h at RT. After washing with PBS (-/-) twice, spheroids were permeabilized with increasing concentrations of methanol (50%, 80% and 100%) at 4°C for 10 min each. Furthermore, samples were washed in different solutions for 10 min each at RT: 20% dimethyl sulfoxide (DMSO) in methanol, 80% methanol following 50% methanol, a PBS wash and a final wash with PBS containing 0.2% Triton. Subsequently, spheroids were incubated in HISTO Penetration buffer (Visikol, USA) for 30 min and blocked in HISTO Blocking Buffer (Visikol, USA) for 60 min at 37°C. Primary antibodies were diluted in HISTO Antibody Buffer (Visikol, USA) and incubated over night at 4°C. On the next day, spheroids were washed 5 times for 10 minutes in HISTO Washing Buffer (Visikol, USA) and incubated with the secondary antibody in HISTO Antibody Buffer containing 4′,6-diamidino-2-phenylindole (DAPI; Thermo Fisher Scientific, USA) for 1 h at RT. Spheroids were washed 10 times for 5 min and cleared with HISTO-M clearing buffer (Visikol, USA). For confocal analysis, cleared spheroids were transferred into a 96-well glass-bottom plate (PerkinElmer, Germany). Pictures were taken with the Leica SP 8 confocal microscope. Spheroids were stained for E-cadherin (ab11512, 1:200, Abcam, UK), LYVE-1 (ab14917, 1:100, Abcam, UK), Desmin (RB-9014-PO, Thermo Fisher Scientific, USA), F4/80 (MCA497GA, 1:100, BioRad, USA) and as secondary antibody AF555-goat-anti-rat (A21434, 1:800, Invitrogen, USA) or AF555 goat-anti-rabbit (A21428, 1:800, Invitrogen, USA).

### Bile canaliculi

To stain for bile canaliculi, spheroids were incubated with 4 μM CellTracker Green CMFDA (Thermo Fisher Scientific, USA) and 10 μM Hoechst (Thermo Fisher Scientific, USA) for 45 minutes at 37°C. Spheroids were washed with media, transferred into a glass bottom plate, and imaged with confocal microscopy (Leica SP8).

### Cyp induction and inhibition

To analyze the metabolic capacity, spheroids were incubated with different Cyp inducers for 72 hours, starting 7 days or 14 days post seeding and analyzed by qRT-PCR. To analyze Cyp3a11, 50 μM Pregnenolone 16α-carbonitrile (PCN, Sigma-Aldrich, Switzerland) was used, 15 μM β-napthoflavone (βNF, Sigma-Aldrich, Switzerland) was used to analyze Cyp1a1 as well as Cyp1a2 activity, and 100 μM Phenobarbital (Pb, Roche, Switzerland) is a known inducer for Cyp2b10 and Cyp3a11. PCN and βNF were dissolved in DMSO as a 1000x stock solution, Pb was dissolved directly in the cell culture media.

For repeated Cyp induction, 7-day old spheroids were incubated with 50 μM Dexamethasone (Sigma-Aldrich, Switzerland), 25 μM Rifampicin (Sigma-Aldrich, Switzerland), or vehicle control for 72 hours. Assessment of Cyp induction was performed using the P450-Glo™ CYP3A4 Assay and a Luciferin IPA substrate (Promega, USA) according to the manufacturer’s instruction. In short, spheroids were washed with medium, and fresh medium containing Luciferin IPA was added to each well and incubated for 60 min at 37°C. After incubation, 25 μl of medium were transferred into a white opaque plate, and 25 μl Luciferin Detection Reagent (Promega, USA) were added and incubated for 20 min at RT. The luminescence signal was measured using a Cytation5 (Biotek, USA). The induced spheroids were cultivated for another week with daily medium changes. On day 14, the same spheroids were induced and measured a second time. To measure potential downregulation of Cyp activity, non-induced spheroids were treated with 10 μM Ketokanazole (Sigma-Aldrich, Switzerland), together with the Luciferin IPA substrate, for 60 min and processed as described above.

### Gene-expression analysis

RNA was isolated from 8 pooled spheroids using the microRNA Micro kit (Qiagen, Germany) and transcribed into cDNA using the First Strand Transcription kit (Roche, Switzerland) according to manufacturer’s instructions. Amplifications were performed on a LightCycler Real-Time Polymerase Chain Reaction System (Roche, Switzerland) using TaqMan®Gene Expression Assays (Applied Biosystems, USA) and TaqMan® Fast Universal PCR Mastermix (Applied Biosystems, USA). Data were normalized to the endogenous control (average of GAPDH and TBP) to assess relative gene expression based on the 2^(−ΔΔCT)^ method [29]. All endogenous control genes were measured in all samples. Data were expressed as fold changes compared to the corresponding untreated control except Fig 4B, were data was compared to monoculture spheroids. Primers included: Cyp3a11 (Mm00731567_m1), Cyp2b10 (Mm01972453_s1), Cyp1a1 (Mm00487218_m1), Cyp1a2 (Mm00487224_m1), F4/80 (Mm0080529_m1), Desmin (Mm00802455_m1) Lyve1 (Mm0080529_m1), F4/80 (Mm0080529_m1), Desmin (Mm00802455_m1), Lyve1 (Mm00475056_m1), BSEP (Mm00445168_m1), Acta2 (Mm01546133_m1), H2-Ab1 (Mm00439216_m1), IL-6 (Mm00446190_m1) and TNFα (Mm00443258_m1). GAPDH (Mm99999915_g1) and TBP (Mm01277042_m1) were used as endogenous control.

### Spheroid treatment and cytotoxicity assay

Compounds were obtained from Sigma-Aldrich if not otherwise stated. Stock solutions of Tetracycline, Diclofenac, Methotrexate, Cyclophosphamide, Acetaminophen, Propranolol and Buspirone, were prepared in DMSO and diluted in maintenance medium to a maximal final DMSO concentration of 0.5%. Toxicity treatment started on day 7 with re-dosing every second day. On the first day of exposure, 50 μl of the cell culture media was removed and 50 μl of fresh cell culture media containing the compound solution was added to the well to achieve the desired concentration. For redosing, 50 μl of cell culture media was replaced with fresh compound-containing cell culture media. The cellular ATP content was measured by using the CellTiter-Glo® 3D Cell Viability Assay (Promega, USA) according to the manufacturer’s instructions. The dose-response curves were plotted, and IC50 values were calculated using Graph-Pad Prism.

### Steatosis

Coculture spheroids were treated for 48 h with 5 μM or 25 μM Cyclosporine A (CsA, Sigma-Aldrich, Switzerland). After exposure, spheroids were fixed with 4% PFA and stained with HCS LipidTox Green neutral lipid stain (1:500, Thermo Fisher Scientific, USA) and DAPI. Confocal images (Number of planes: 100, Distance: 0.5μm) were taken from 8 individual spheroids per condition in glass-bottom plates with the PerkinElmer Opera Phenix. The Harmony 4.8 Software was used for the quantification of neutral lipid accumulation by normalizing the amount of lipids to the area of the corresponding spheroid.

### Cholestasis

On day 7 post seeding, coculture spheroids were treated with different concentrations of Chlorpromazine (CPZ, Sigma-Aldrich, Switzerland) and Troglitazone (LKT Labs, USA) for 24 h. Bile salt export pump (BSEP) downregulation was measured from pooled spheroids via qRT-PCR.

### Statistical analysis

Prism software (Graph Pad Software) was used to analyze the generated data and to determine statistical significance between treatment groups by applying the unpaired Student’s t-test. P values of < 0.05 were considered to be significant.

## Results and discussion

### Mouse liver spheroids are viable and functionally active over three weeks

**Fig 1:**
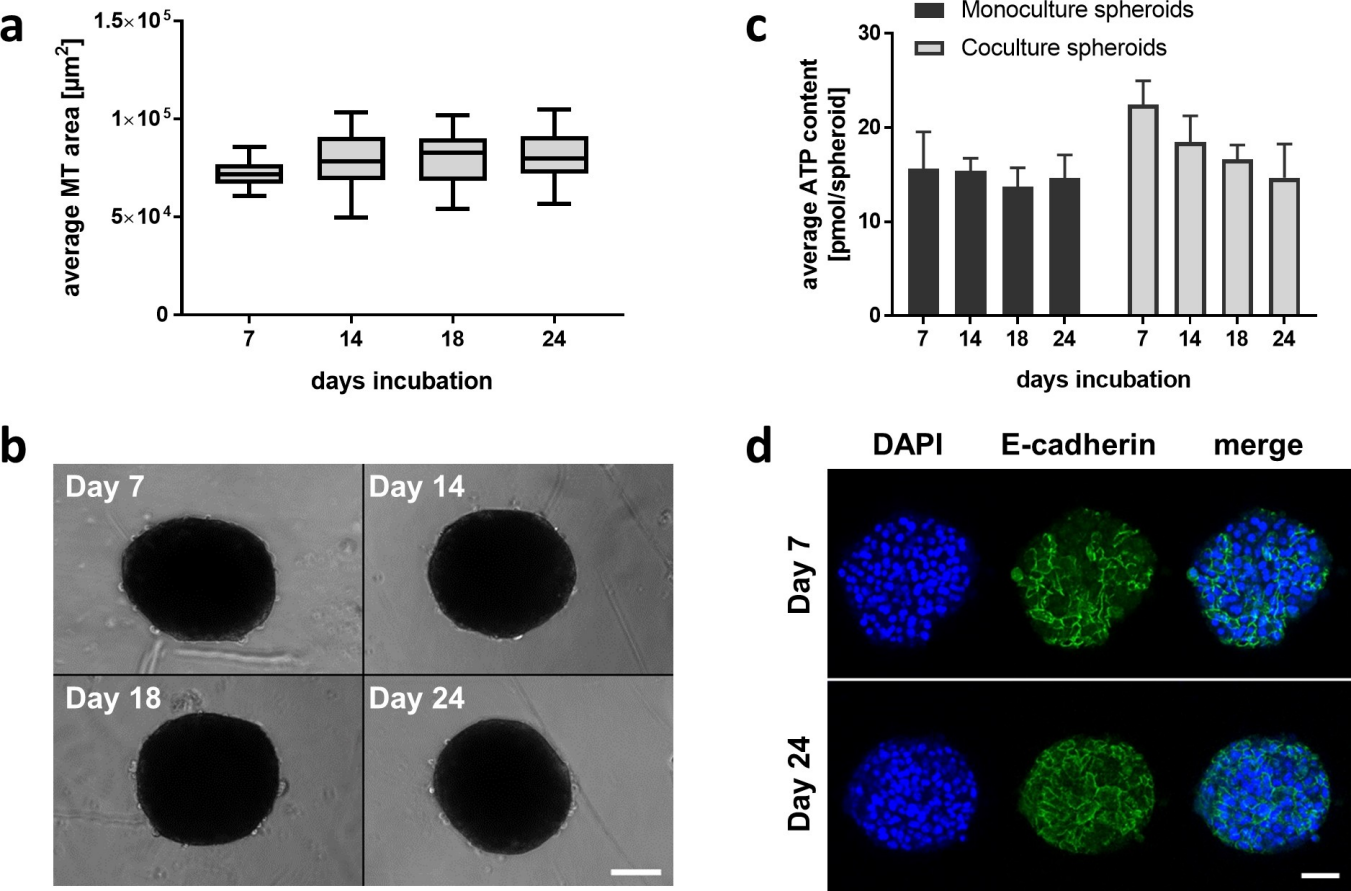
Viability and morphology of liver 3D spheroids during three weeks of continuous culturing. **a** Size analysis of hepatocyte spheroids cultured for 24 days. Data is shown as multiples of the size at day 7, represented as average ± standard deviation (n = 16 spheroids per time point) **b** Bright-field images of spheroids on day 7, 14, 18, and 24. Scale bar = 50 μm. **c** Average ATP content of liver spheroids, cultured over 24 days, data represented in pmol/spheroid ± standard deviation (n = 16 spheroids per time point) **d** E-cadherin staining showing cell-cell interactions of hepatocytes at day 10 and day 24 post seeding, scale bar = 50 μm.

To assess long-term stability and liver-specific functions of the murine spheroids, we assessed different parameters, including morphology, viability and functionality over a period of three weeks. A mouse hepatocyte is larger than a rat or human one (mouse > rat > human), which had to be considered in determining the number of cells to be used for spheroid generation [30–32]. Testing of different cell numbers per spheroid resulted in an optimal spheroid consisting of 1250 hepatocytes with an average diameter of ~250–300 μm (S1 Fig). This size allows for sufficient nutrient and oxygen supply in the center of the spheroid, which helps to avoid the development of a necrotic core [33]. We used ultra-low attachment round-bottom well plates for forming the spheroids. After seeding and centrifugation, the cells accumulated in the center of the wells, and spheroids were formed in approximately 7 days (S2A Fig). On day 4, the culture medium was gradually replaced with FBS-free maintenance medium to enable long-term cultivation in low-serum conditioned medium. Note, although the serum is gradually diluted in the cell culture medium by replacing 50% of the medium with serum free medium, it cannot be ruled out that traces of serum remain in the well or stick to the surface of the culture plate. Therefore, we use the term low-serum. In our case, the low-serum conditions allowed to better reflect the drug exposure of the cells, since the exposure can be altered with strong serum binding drugs under high serum condition. The size of the spheroids was found to be consistent with a diameter of ~250–300 μm with sharp and defined boarders during the whole culturing period of three weeks (Fig 1A and 1B). Compared to other studies, we did not see a decrease in size over time [12]. In addition, nuclear staining after 3 weeks in culture shows no sign of nuclear blebbing or fragmentation (S2b Fig). Cell viability was measured for both mono- and coculture spheroids by assessing intracellular adenosine-triphosphate (ATP) content, which was sustained over three weeks of culturing (Fig 1C). In monoculture spheroids, we detected stable ATP levels from day 7 to day 24. The ATP level in coculture spheroids were ~ 40% higher on day 7, when compared to monoculture spheroids, which correlates with the addition of the NPCs in the spheroid. Over the 3-week culture period, the ATP level of the coculture spheroids decreased from 20.52 ± 2.54 pmol/spheroid on day 7 to 14.69 ± 3.56 pmol/spheroid on day 24. This might indicate that NPCs or hepatocytes are impacted negatively by the coculture leading to apoptosis of some cells. Nevertheless, the ATP contents show viable liver spheroids and the values are in concordance with previously published data [12, 16]. To determine cell-cell interactions, an E-cadherin staining was performed on day 10 and day 24, which showed consistent expression in murine liver spheroids (Fig 1D).

**Fig 2:**
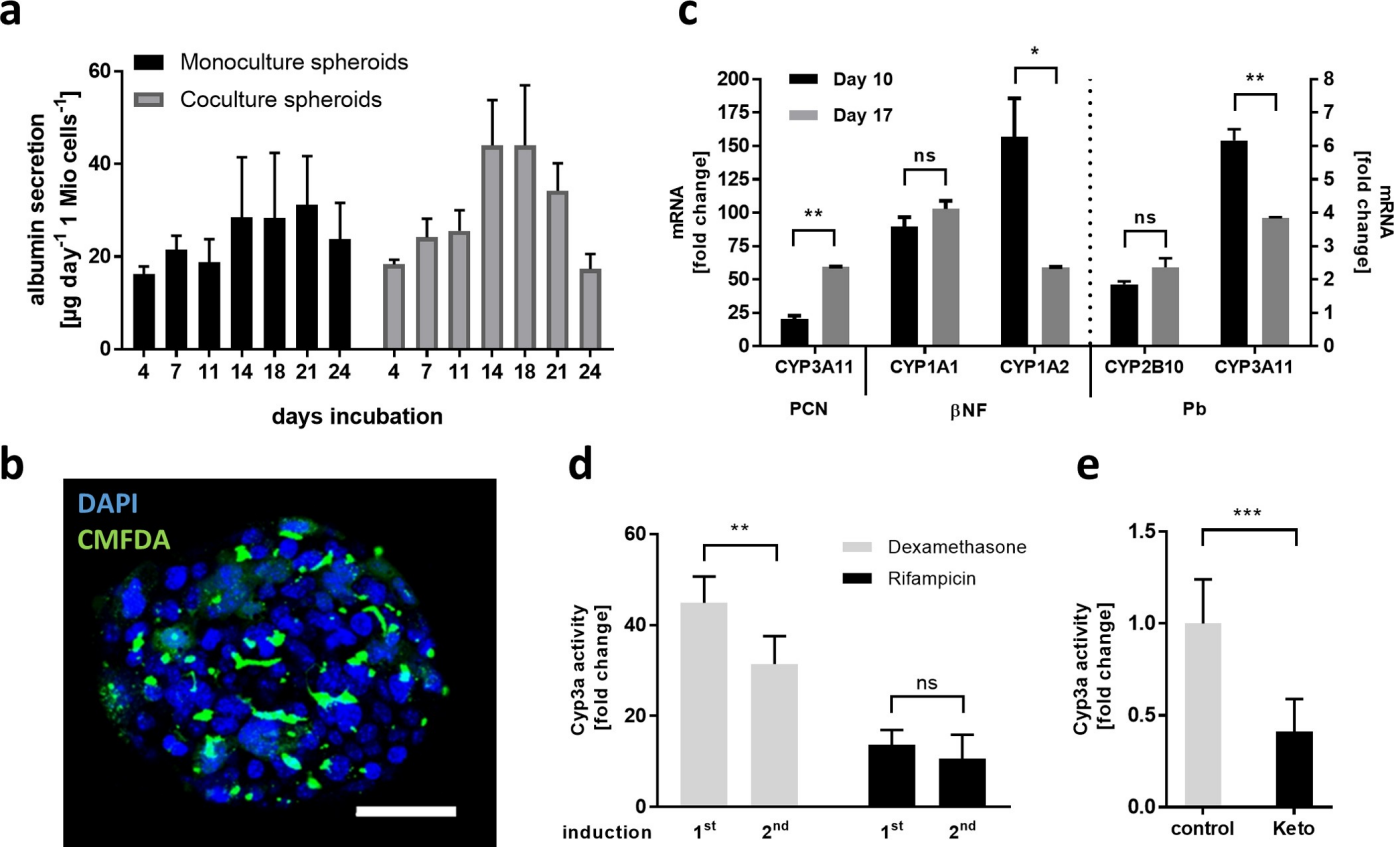
Metabolic activity of liver spheroids is maintained over three weeks. **a** Average albumin release of liver spheroids, cultured for 24 days, data represented as μg day^-1^ 1 Mio cells^-1^ ± standard deviation (n = 16 spheroids per time point) **b** CMFDA (green) accumulation showing bile canaliculi in 10-day old spheroids with DAPI (blue), scale bar = 100 μm. **c** Cyp450 induction of spheroids treated for 3 days on day 7–10 (black bar) and day 14–17 (grey bar) with 50 μM Pregnenolone 16α-carbonitrile (PCN), 15 μM β-naphthoflavone (βNF), and 100 μM Phenobarbital (Pb). Gene expression was measured via qRT-PCR, data is represented as multiples of the change in the vehicle control ± standard deviation (n = 4) **d** Cyp450 activity measured in the supernatant of the same spheroids on day 7–10 (1^st^ induction) and day 14–17 (2^nd^ induction) with 50 μM Dexamethasone (grey bar) and 25 μM Rifampicin (black bar). Data shown as multiples of the change in the vehicle control ± standard deviation (n = 8 spheroids per condition) **e** Cyp3a inhibition with Ketoconazole (Keto), measured via a Cyp450 activity assay after 48 h incubation. Data represented as multiples of the change in the vehicle control ± standard deviation (n = 8 spheroids per condition). Statistical significance is indicated * (p-value ≤ 0.05; Student’s t-test), ** (p-value ≤ 0.01; Student’s t-test), and *** (p-value ≤ 0.001; Student’s t-test), ns meant not significant (p-value > 0.05).

The production and secretion of albumin into the bloodstream is one of the major functional parameters of the liver. Measuring albumin secretion in the supernatant was, therefore, assessed during the whole culturing period (Fig 2A). Monoculture spheroids showed a stable secretion of albumin with higher variability starting day 14 after spheroid production. coculture spheroids secreted similar amount of albumin, although an increase was detected starting 14 days after production (19 ± 5 μg day^-1^ 1Mio cells^-1^ on day 11 to 44 ± 10 μg day^-1^ 1Mio cells^-1^ on day 14) following a continuous decline to values detected in the first week after production. In addition to the albumin secretion, we have monitored Cyp3a activity in both mono- and coculture spheroids, which showed stable basal activity over the culture period of 3 weeks in monoculture spheroids (S3 Fig). Coculture spheroids, on the other side, showed declining Cyp3a activity in the first days before stabilizing to around 60% compared to day 4. We can see that the additional NPCs have an impact on the functionality of the hepatocytes, which is not surprising in a quite complex coculture system, particularly when cultured as spheroids. The cytoarchitecture of liver cells in the spheroids hardly resemble the situation *in vivo*, as they are randomly distributed throughout the spheroid. *In vivo*, these cells promote both homotypic and heterotypic cell-cell contacts and their signaling is important for homeostasis. This signaling might not, or to a lesser extent, be achievable in a spherical structure. Therefore, NPCs contributed to the fluctuation of albumin release and to the downregulation of Cyp3a activity in coculture spheroids.

In summary, considering functionality of hepatocytes, coculture spheroids showed higher fluctuations compared to monoculture spheroids, although functionality was maintained in both systems over the culture period of 3 weeks.

Another unique function of the liver is the production and secretion of bile. To assess the spontaneous development of bile canaliculi within the spheroids and the capacity of the hepatocytes to secrete bile, we incubated the spheroids with the fluorescent marker CMFDA, which diffuses into the hepatocytes and is actively transported into the bile canaliculi. By fluorescence imaging, we could detect accumulation of CMFDA in the spheroids, indicating that the hepatocytes are indeed able to take up and transport bile acids into the channels (Fig 2B).

For the biotransformation of endogenous compounds and xenobiotics including drugs, liver cytochromes P450 (Cyp450) play a critical role as they convert substances into more polar products to facilitate the elimination from the body. The major drug-metabolizing Cyp450 in mouse include Cyp1a1, Cyp1a2, Cyp2b10, and Cyp3a11 [34]. To assess their metabolic capacity, spheroids were incubated with well-known Cyp450 inducers for 48 hours and analyzed for Cyp450 induction on RNA level on day 10 and day 17 (Figs 2C and S4). All tested drugs induced the specific Cyp450, both on day 10 and on day 17, although differences in the extend of induction was visible. Pregnenolone 16α-carbonitrile (PCN) is a well-known Cyp3a11 inducer and produced a 20-fold induction on day 10 and a significantly higher induction (59-fold) on day 17 compared to the vehicle control. When treated with β-naphthoflavone (βNF), a strong Cyp1a inducer, both Cyp1a1 and Cyp1a2 mRNA were induced 90-fold and 157-fold on day 10, and 103-fold and 59-fold on day 17, respectively. Here, although similar induction potential of Cyp1a1 mRNA on day 10 and 17 could be detected, a significantly lower induction in Cyp1a2 mRNA was reported after 2 weeks in culture. Additionally, spheroids were incubated with Phenobarbital (Pb), a known Cyp2b10 and Cyp3a11 inducer. With this treatment, Cyp2b10 mRNA was induced 1.9-fold on day 10 and 2.4-fold induced on day 17, both times showing a moderate but significant induction. Additionally, Pb induced Cyp3a11 mRNA 6.2-fold on day 10 and a significantly lower induction (3.9-fold) on day 17.

Taken into consideration that drugs are often administered repeatedly, we challenged the same spheroids with repeated dosing of Dexamethasone and Rifampicin in order to see if Cyp450 can be induced multiple times. As shown in Fig 2D, Cyp3a induction was possible on day 7–10 as well as re-induction on day 14–17. Dexamethasone is known for its ability to strongly induce Cyp3a in mouse, as seen by the 45-fold induction at day 7–10. The second stimulation still led to a 31-fold induction of Cyp3a compared to the unstimulated control. This induction, however, was significantly lower than the first induction on the earlier time point. Rifampicin is reported as weaker inducer, showing 14-fold and 11-fold induction, respectively [35]. Here, no difference between the time points could be observed, which might indicate that repeated inducibility of Cyp3a is reduced only at high induction. In addition to Cyp450 induction, inhibition of Cyp450 activity by drugs may have an impact on the metabolism of co-administered drugs. We, therefore, also assessed the Cyp3a activity upon treatment with Ketoconazole, a known Cyp3a inhibitor [36]. As shown in Fig 2E, the Ketoconazole treatment resulted in a significantly downregulation of Cyp3a activity by 60%.

In Summary, we have shown that primary mouse hepatocyte spheroids can be cultured over a period of three weeks in low-serum media. The spheroids remained functionally active in terms of albumin production, presence of bile canaliculi, and metabolic capacity.

### Mouse hepatocyte spheroids can be used to predict the hepatotoxic potential of a set of model compounds

**Fig 3:**
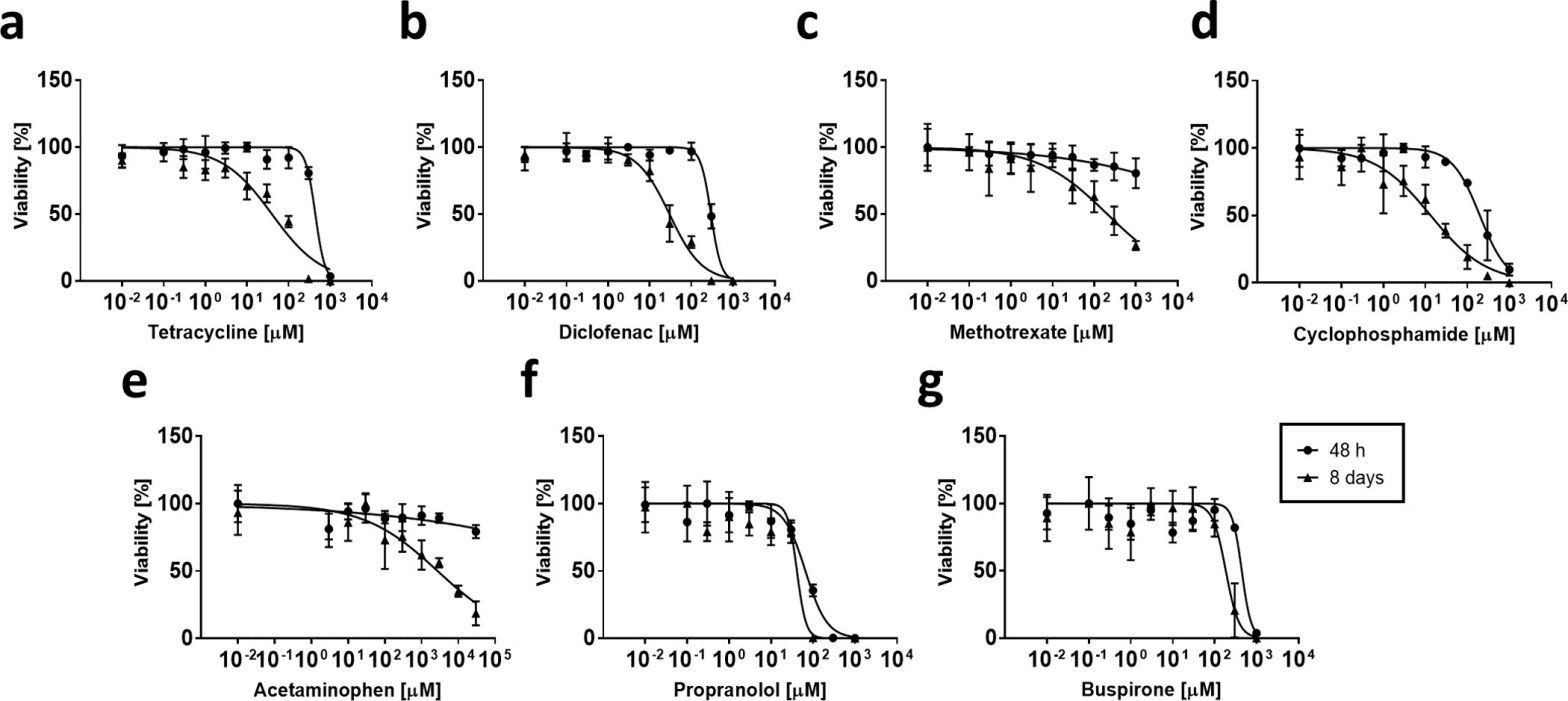
Comparison of short-term and long-term toxicity profiles of known DILI and non-DILI compounds in liver spheroids. Liver 3D spheroids were treated with a single dose during 48 h or with repeated dosing during 8 days with different concentrations of Tetracycline (**a**) Diclofenac (**b**) Methotrexate (**c**) Cyclophosphamide (**d**) Acetaminophen (**e**) Propranolol (**f**) and Buspirone (**g**). Spheroids were treated once during 48 h with a single dosage (circle), or repeatedly every 2nd day during 8 days in total (triangle) starting at day 7 post seeding. Viability was determined by measuring ATP content (n = 4 spheroids per concentration and time point), and the data are represented as % average in reference to vehicle control ± standard deviation.

Drug-induced liver injury (DILI) is a major concern in drug development, which often cannot be predicted *in vitro* due to inherent limitations of 2D hepatocyte cultures, including short-term viability and decreased functionality. Using a sophisticated hepatocyte model, long-term dose-response testing of molecules can help to identify hepatotoxic compounds early on, can enable the understanding of the mechanism of toxicity, and can help to better define rodent *in vivo* studies. Here, we tested the long-term exposures of spheroids consisting of parenchymal cells with the known hepatotoxic agents Tetracycline, Diclofenac, Methotrexate, Cyclophosphamide, and Acetaminophen as well as non-DILI compounds Propranolol and Buspirone and compared the results to known mouse *in vivo* data (Fig 3 and Table 1). The spheroids either experienced a single treatment and were analyzed after 48 hours (Fig 3A–3G, 48 h), or they were repeatedly treated every 2 days and analyzed at day 8 (Fig 3A–3G, 8 days). In both cases, ATP content was used as a viability marker. Cytotoxicity was assessed by measuring the IC_50_ value, the compound concentration that is lethal to 50% of the cells (Table 1).

**Table 1:** IC_50_ values of DILI and non-DILI compounds in 3D liver spheroids as well as reported mouse C_max_ values (n.a.: Not available).

Compound	3D *in vitro* IC_50_ [μM]		Mouse C_max_ [μM] ^(ref)^
	48 h	8 days	
Tetracycline	436.1	39.7	11 [37]
Diclofenac	294.0	29.7	10 [38]
Methotrexate	389’989	206.7	43 [39]
Cyclophosphamide	197.1	13.29	77 [40]
Acetaminophen	644’301’371	2750	2216 [41]
Propranolol	65.84	41.3	n.a.
Buspirone	442.7	184.4	n.a.

The IC_50_ values of Tetracycline (Fig 3A), Diclofenac (Fig 3B), and Cyclophosphamide (Fig 3D) were 10-fold lower upon repeated treatments as compared to single treatments. Interestingly, the corresponding concentrations correlate with the maximal plasma concentration (C_max_) (Table 1), reported from *in vivo* mouse studies, which indicates that the use of spheroids might be suitable for recapitulating *in vivo* hepatotoxic outcomes [37, 38, 40]. Methotrexate (Fig 3C) and Acetaminophen (Fig 3E) showed only a slight reduction in cell viability after a single dosage, which resulted in high IC_50_ values. In comparison, re-dosing resulted in lower IC_50_ values, also in concordance with previously reported mouse *in vivo* C_max_-values [39, 41]. Propranolol and Buspirone yielded a rather stable dose-response curve with similar IC_50_ values upon single or repeated dosing (Fig 3F and 3G).

To compare the results to human *in vivo* concentrations, Table 2 represents the ratio of mouse *in vitro* IC_50_-values and human *in vivo* C_max_-values, which is used to determine the margin of safety (MOS). Compounds were assigned to the class of (i) hepatotoxic agents, if the IC_50_/C_max_ value was lower than 100 and to (ii) non-hepatotoxic agents, if the value was higher than 100 [42]. From the substances tested, Tetracycline, Diclofenac, and Cyclophosphamide were flagged as hepatotoxic agents already after 48 hours, while Methotrexate and Acetaminophen were only assigned to the correct class after repeated exposures. As expected, Propranolol and Buspirone were assigned correctly as non-DILI compounds in both short-term and long-term exposure experiments.

**Table 2:** Classification of hepatotoxicity using the margin of safety (MOS) by comparing *in vitro* IC_50_ to human C_max_ values.

Compound	Human C_max_ [μM] ^(ref)^	MOS [3D *in vitro* IC_50_ / Human C_max_]	
		48 h	8 days
Tetracycline	20.96 [43]	**20.81**	**1.89**
Diclofenac	9.43 [42]	**31.18**	**3.15**
Methotrexate	4.63 [44]	*84’231*	**44.64**
Cyclophosphamide	96 [45]	**2.05**	**0.14**
Acetaminophen	136 [46]	*4’737’510*	**20.22**
Propranolol	0.2 [47]	*329*	*207*
Buspirone	0.009 [48]	*49189*	*20489*

^**< 100 = hepatotoxic,** > 100 = non-hepatotoxic.^

This data set shows the importance of model systems that allow for repeated drug exposure experiments for DILI assessment, which was achieved by using 3D hepatocyte spheroids. Although for some compounds, like acetaminophen, 2D cell cultures and single dosing can be used for detecting acute toxicity [49], 3D culture systems have shown to enable better predictions of the acute and chronic DILI potential compared to traditional culturing of hepatocytes [50–53]. For acetaminophen, repeated dosing of the mouse hepatocyte spheroids revealed an IC_50_ value similar to *in vivo* data. However, *in vivo*, acute toxicity is already seen in the first 24 hours of an overdose. Acetaminophen is metabolized by CyP450 into the highly toxic product N-acetyl-p-benzoquinone imine (NAPQI), a reactive metabolite which is detoxified by binding to reduced glutathione (GSH). At toxic concentrations, glutathione is depleted and NAPQI can bind to intracellular proteins, RNA, and DNA, which subsequently leads to hepatocyte necrosis [54]. The reason for the observed delayed toxicity in the 3D hepatocyte spheroids will need further investigations. Furthermore, the partial redosing of the spheroids (50% removal, 50% addition of fresh media containing the desired concentration) can lead to an accumulation and upconcentration of byproducts, as these byproducts cannot be eliminated (e.g. by the kidney) which could influence the viability of the hepatocytes.

In summary, with the long-term exposure schedule we were able to obtain similar IC_50_ values seen in mouse *in vivo* studies, and correctly classified all seven selected compounds as hepatotoxic or non-hepatotoxic molecules. Therefore, the spheroids constitute a suitable model to reproduce and investigate DILI findings detected in mouse *in vivo* studies. To use the spheroid model as screening system for early DILI assessment, additional testing of hepatotoxic and non-hepatotoxic molecules, which feature different mechanism of toxicity, will be necessary to fully validate the system.

### Primary liver spheroids can be cultured with parenchymal and non-parenchymal cells

**Fig 4:**
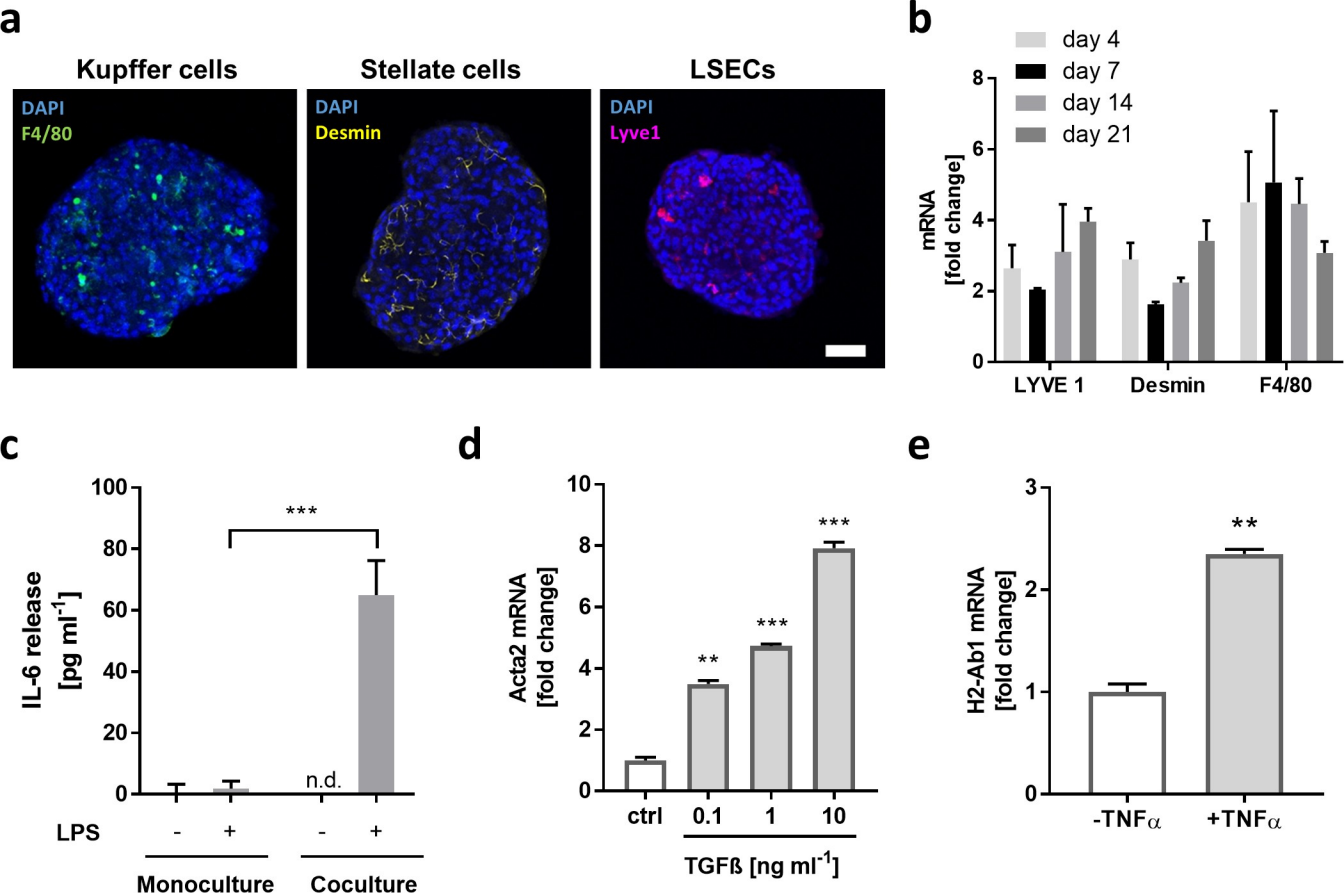
Coculture spheroids representing features of the innate immune system. **a** Immunofluorescence staining in mouse 3D liver spheroids 14 days after seeding revealed the presence of Kupffer cells (F4/80), stellate cells (Desmin) and liver sinusoidal endothelial cells (LYVE-1). Scale bar = 50 μM. **b** qRT-PCR data of NPC markers on day 4, 7, 14, and 21 after seeding. Data is represented as x-fold change compared to monoculture spheroids ± standard deviation (n = 4) **c** IL-6 release of monoculture and coculture spheroids treated with 10 μM (+) or without (-) LPS for 72 h 14 days after production (n.d., not detected, signifies values below the detection limit) (n = 8 spheroids per condition). **d** Induction of Acta2 mRNA (alpha smooth muscle actin) in liver coculture spheroids treated with different concentrations of TGFβ for 72 h. Data is represented as x-fold change in reference to the vehicle control (ctrl) ± standard deviation (n = 4) **e** Induction of H2-Ab1(MHC-II) mRNA in coculture spheroids treated with TNFα for 48 h. Data is represented as x-fold induction in reference to vehicle control (-TNFα) ± standard deviation (n = 4). Statistical significance is indicated ** (p-value ≤ 0.01; Student’s t-test), and *** (p-value ≤ 0.001; Student’s t-test).

Hepatocyte monoculture spheroids represent a valuable alternative to 2D cultures to study and monitor long-term drug exposure. However, certain types of DILI can be attributed to the development of immune responses that ultimately lead to non-viable hepatocytes. To capture such immune-mediated liver toxicity, a more complex *in vitro* liver model including non-parenchymal cells (NPCs) would be required. We, therefore, established coculture spheroids including (i) Kupffer cells (KCs), the main functions of which include phagocytosis and defense of the liver against bacteria, endotoxins and viral infections; (ii) stellate cells (SCs), which are the most important cell type involved in fibrosis; (iii) and liver sinusoidal endothelial cells (LSECs), which are involved in endocytosis, antigen presentation and leukocyte recruitment [55–58]. The cell fractions in the liver include approximately 60% hepatocytes, 16% LSECs, 12% KCs, and 8% SCs and 4% other cell types [59–61]. During cell purification, the NPC fraction was treated as a whole, meaning that the fractions of the different NPC subtypes remained untouched. To achieve a physiological ratio, we formed the coculture spheroids with a number 2:1 ratio by using 1250 parenchymal and 650 non-parenchymal cells. Immunofluorescence staining on day 14 revealed the presence of KCs (F4/80), SCs (Desmin), and LSECs (LYVE-1) distributed evenly over the spheroid (Fig 4A). When monitoring the presence of NPCs through gene expression over time, we detected similar mRNA levels for all cell types on day 4, 7, 14, and 21 (Fig 4B). The mRNA level of LSECs showed a slight increase over time, ranging from 2.6-fold on day 4 to 4-fold on day 21, which might indicate a proliferative potential of LSECs. The mRNA expression levels of SCs were stable over time (day 4 2.9-fold ± 0.5, day 7 2.7-fold ± 0.1, day 14 2.2-fold ± 0.1 and day 21 3.4-fold ± 0.6) and the KCs levels show a slight decrease on day 21 (day 4 4.5-fold ± 1.4, day 7 5.1-fold ± 2, day 14 4.5-fold ± 0.7, and day 21 3.1-fold ± 0.3).

NPCs are activated upon detection of endotoxins, such as lipopolysaccharides (LPS), which leads to the secretion of certain cytokines by NPCs [62]. To assess the functionality and activation potential of NPCs, monoculture and coculture spheroids were treated with LPS for 72 hours and analyzed for interleukin 6 (IL-6) release (Fig 4C). The monoculture spheroids showed only little induction of IL-6 (2 pg ml^-1^), which indicates that the monoculture spheroids are free of NPCs. As expected, the coculture spheroids showed high IL-6 release (65 ± 11 pg ml^-1^) and no IL-6 release if not stimulated with LPS. These results confirm not only the presence of NPCs in the coculture spheroids but also their quiescent state and the possibility to activate these cells in our 3D *in vitro* system. Stellate cells are quiescent in a normal liver but can be activated upon liver damage and are responsible for scar-tissue formation. Stimulation with transforming growth factor beta (TGFβ), triggered a dose-dependent induction of Acta2 (alpha smooth muscle actin, αSMA), which is an activation marker for stellate cells (Fig 4D). Low concentrations of TGFβ increased Acta2 induction by 3.5 fold, whereas higher concentrations induced gene expression by 4.7-fold and 8-fold, respectively. The functional characterization of liver sinusoidal endothelial cells was performed by induction of the major histocompatibility complex II (MHC-II), which is constitutively expressed in LSECs but can be further increased by exposure to pro-inflammatory cytokines, such as tumor necrosis factor alpha (TNFα) [63]. Fig 4E shows that TNFα can increase the mRNA expression of MHC-II by 2.3-fold.

Taken together, these results demonstrate the potential of using liver coculture spheroids containing the four major cell types from the same donor without losing non-parenchymal cells over time. Kupffer cells, stellate cells, as well as liver sinusoidal endothelial cells are functionally active, which we demonstrated by their activation using different stimuli.

### Interaction of parenchymal and non-parenchymal cells can be recapitulated in 3D liver spheroids

**Fig 5:**
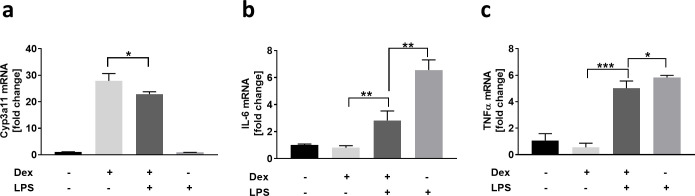
Interaction of parenchymal and non-parenchymal liver cells. Liver coculture spheroids were treated for 48 h with either vehicle control (-/-), 50 μM Dexamethasone (+/-) or 10 μg/ml LPS (-/+) alone, or 24 h with LPS following 24 h with LPS and Dexamethasone (+/+). Gene expression of Cyp3a11 (**a**) IL-6 (**b**) and TNFα (**c**) was measured via qRT-PCR. Data presented as average x-fold change in reference to vehicle control ± standard deviation (n = 4). Statistical significance is indicated * (p-value ≤ 0.05; Student’s t-test), ** (p-value ≤ 0.01; Student’s t-test), and *** (p-value ≤ 0.001; Student’s t-test).

Drug-induced liver injuries can be caused by several modes of action, including perturbations of biochemical pathways caused by the interaction of parenchymal and non-parenchymal cells. These complex and dynamic cellular interactions can only be represented *in vivo* or in coculture models, which include the parenchymal and non-parenchymal cells. To assess the interplay of the different cell types in our liver spheroids, we investigated the influence of liver inflammation on Cyp3a11 mRNA in hepatocytes (Fig 5). NPC activation upon LPS stimulation and subsequent cytokine release have been shown to affect gene expression levels and the function of Cyp450s [64, 65]. Therefore, we treated healthy (- LPS) and inflamed (+ LPS) liver spheroids with Dexamethasone and analyzed Cyp3a11 induction on mRNA level (Fig 5A). In healthy liver spheroids (- LPS), Dexamethasone treatment increased Cyp3a11 mRNA expression by 28-fold in comparison to the non-stimulated control. Interestingly, the mRNA expression was reduced by 18% (p = 0.004) if spheroids were in an inflamed state (+ LPS), whereas LPS alone did not lead to Cyp3a11 mRNA induction. The anti-inflammatory effect of the glucocorticosteroid Dexamethasone on the activation potential of the liver non-parenchymal cells was also investigated and is shown in Fig 5B and 5C. As expected, LPS alone increased IL-6 mRNA expression 6-fold, whereas co-administration of Dexamethasone reduced the induction by ~50% (Fig 5B). Gene expression of TNFα was also affected through the addition of Dexamethasone, however yielded only moderate reduction as compared to IL-6 (Fig 5C).

The induction of Cyp450 is regulated by the binding of xenobiotics to nuclear receptors, such as aryl hydrocarbon receptor (AhR), constitutive androstane receptor (CAR), and pregnane X receptor (PXR). Dexamethasone is a specific PXR agonist, which binds to the receptor and causes its translocation to the nucleus where it initiates the transcription of Cyp3a11. During inflammatory response, here triggered by LPS, activated KCs and LSECs release IL-6 which binds to the IL-6 receptor expressed on hepatocytes. This IL-6/IL-6R complex leads to phosphorylation and the activation of multiple signaling cascades, also resulting in the decrease of PXR gene expression, which consequently leads to lower Cyp3a11 mRNA induction when treated with Dexamethasone [66].

The downregulation of Cyp3a11 upon liver inflammation has been demonstrated in mouse *in vivo* studies [64, 67, 68], and has also been shown in rat and human liver models that include the hepatocytes and Kupffer cells [69–71]. Here, we show for the first time, that also 3D mouse liver spheroids are capable of recapitulating this complex interplay of parenchymal and non-parenchymal cells by using Dexamethasone as an example molecule. In the mouse *in vivo* study of Moriya et al, the PXR agonist pregnenolone-16α-carbonitrile (PCR), showed a 25% reduced expression of Cyp3a11 mRNA when administered together with LPS [64], which is in concordance with our data generated *in vitro*.

Taken together, we could show the strong interplay between non-parenchymal and parenchymal cells and their effect on liver metabolism as well as the anti-inflammatory effect of Dexamethasone with our mouse 3D liver spheroids, which, again demonstrates the physiological relevance of the developed *in vitro* system.

### Onset of liver diseases, such as steatosis or cholestasis, can be recapitulated in murine 3D liver spheroids

**Fig 6:**
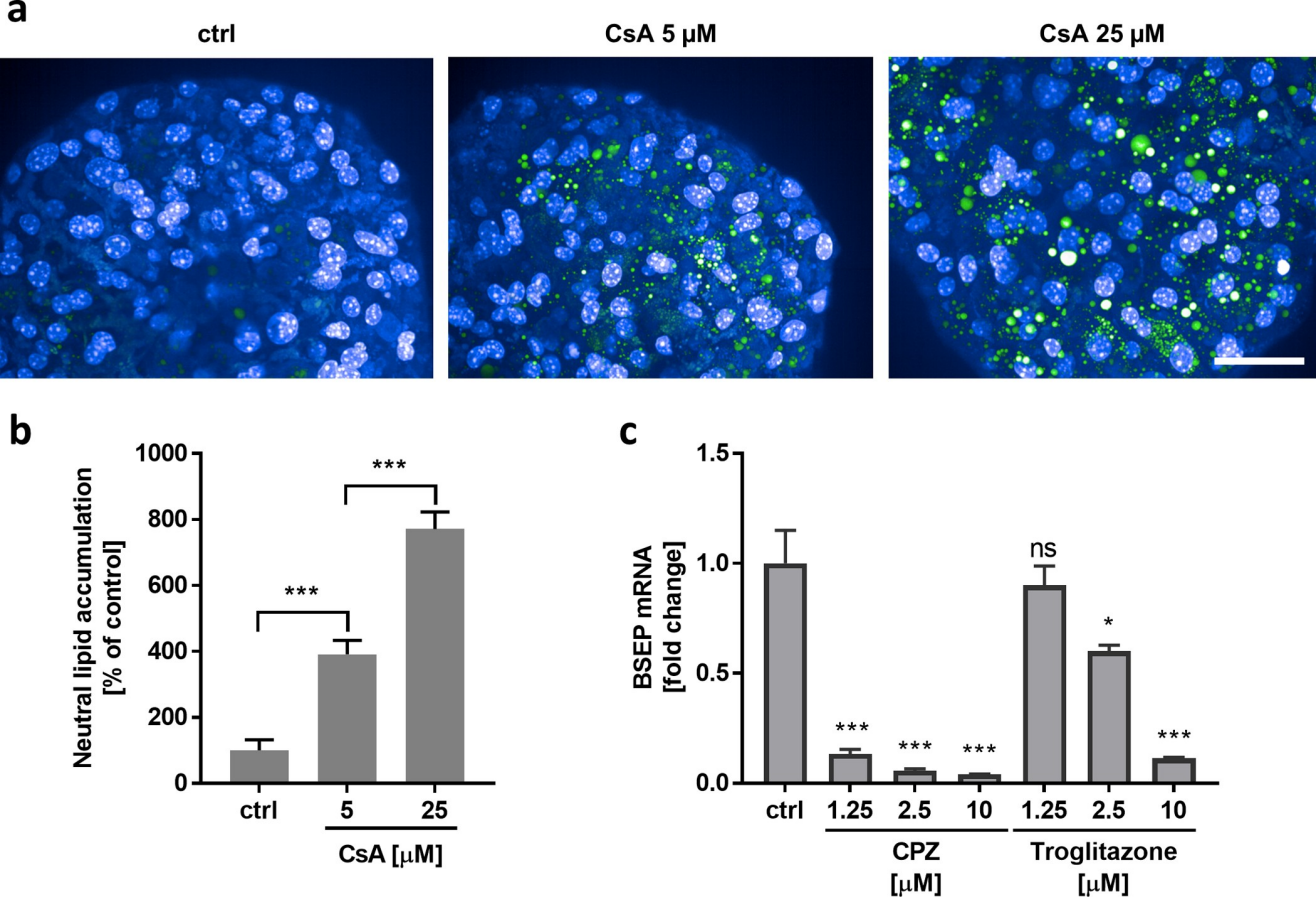
Drug-induced steatosis and cholestasis in 3D liver spheroids using Cyclosporine A, Cyclophosphamide, and Troglitazone as model compounds. **a** Coculture spheroids were treated for 48 h with 5 μM and 25 μM Cyclosporine A (CsA) and stained with DAPI (blue) and for neutral lipids (green) using immunofluorescence. Scale bar = 50 μm **b** Quantitative analysis of neutral-lipid accumulation in CsA-treated spheroids. Data are represented as accumulation in % with respect to vehicle control (ctrl) ± standard deviation. (n = 8 spheroids per condition). **c** Coculture spheroids were treated with different concentrations of Cyclophosphamide (CPZ) and Troglitazone for 24 h and analyzed for BSEP gene expression via qRT-PCR. Data are expressed as x-fold change compared to vehicle control (ctrl) and corrected to GAPDH mRNA ± standard deviation (n = 4). Statistical significance is indicated * (p-value ≤ 0.05; Student’s t-test), ** (p-value ≤ 0.01; Student’s t-test), and *** (p-value ≤ 0.001; Student’s t-test), ns meant not significant (p-value > 0.05).

DILI can be triggered by different mechanisms which can also manifest in liver diseases like steatosis, fibrosis, and cholestasis. Recapitulating these liver-disease phenotypes in spheroids could be used to identify such drugs early in development, further support the study of associated liver diseases, and help developing new drug molecules. Using Cyclosporine A (CsA), Troglitazone, and Chlorpromazine (CPZ), three known DILI compounds, we tested the potential of our coculture spheroids to recapitulate drug-induced steatosis and cholestasis (Fig 6).

Hepatic steatosis is the impairment of normal synthesis and elimination of triglycerides, which results in an abnormal accumulation of lipids in liver cells. Steatosis can further progress into non-alcoholic steatohepatitis (NASH), characterized by hepatocellular injury, inflammation, and even fibrosis, and can ultimately develop into cirrhosis, liver failure, or liver carcinoma [72]. To assess the possibility to induce lipid retention in the liver cells, we treated our liver coculture spheroids with CsA, a compound known to induce hepatic steatosis, for 48 hours and analyzed the lipid content via immunofluorescence (Fig 6). Compared to non-treated control, a slight decrease in cell viability was visible in both treatment groups, however statistically significant cytotoxicity was not observed (ctrl to 5 μM p = 0.6282, ctrl to 25 μM p = 0.2174, student t-test, n = 8 spheroids per condition) (S5 Fig). Only minor levels of lipids could be detected in the control group, which indicated healthy, non-steatotic liver cells in the spheroids during culturing (Fig 6A and 6B). Low concentration of CsA already increased the lipid content to around 400%, compared to the control group, and high concentration of CsA produced even an 8-fold-increased lipid accumulation compared to non-treated spheroids. These findings demonstrate that (i) our 3D model system can be maintained in a non-steatotic condition, and that (ii) the spheroids can be induced to recapitulate steatotic pathologies *in vitro*.

The generation and secretion of bile salts is one of the livers’ key function. If this mechanism is impaired, bile salts remain and accumulate in hepatocytes, which leads to intrahepatic cholestasis and severe liver diseases. One important player in this transport is the bile-salt-export pump (BSEP), the function of which can be inhibited through several factors including drug exposure [73]. To assess the inhibition potential of CPZ and Troglitazone, two known drugs to disturb BSEP function, we incubated the spheroids for 24 hours and analyzed gene expression of BSEP via qRT-PCR (Fig 6C). Compared to the positive control, no cytotoxicity by LDH or ATP was observed under all treatment conditions (S6 Fig). Already low concentrations of CPZ fully inhibited BSEP expression to up to 87%, which increased to 96%, when high concentrations of CPZ were used. Troglitazone, on the other hand, yielded moderate inhibition at low concentrations (10% at 1.25 μM), which increased to 40% (at 2.5 μM) and 89% (at 10 μM), respectively. Compared to CPZ, where mainly the parent compound hampers BSEP function, the metabolite Troglitazone-sulfate strongly inhibits BSEP, which might explain the difference in the extent of BSEP downregulation and further demonstrates the importance of functional active liver cells that are capable of metabolizing compounds like Troglitazone in such an *in vitro* system [74]. In summary, the obtained results indicate that the mechanisms associated to CPZ and Troglitazone-induced cholestasis can be recapitulated in our mouse 3D model system.

Taken together, the 3D liver spheroids can be used to model liver diseases, such as hepatic steatosis or drug-induced cholestasis, and can be used to investigate, better understand, and find treatments against those diseases.

## Conclusion

Our work shows, for the first time, the characterization of a mouse 3D liver spheroid model and demonstrates its physiological relevance in terms of long-term viability and functionality. In preclinical research, mice are among the most commonly used rodent species to investigate the efficacy of novel therapies and, thus, treatment related hepatotoxic effects can already be detected in the early stages of drug development. The mouse 3D liver spheroids presented here are a suitable model system to recapitulate and investigate those toxicity-related issues *in vitro* before addressing them in the human equivalent 3D model. The mouse liver spheroids, have not only the potential to serve as a more physiological relevant liver model, but also constitute an ideal translational bridging tool to address the human relevance of mouse *in vivo* findings. Moreover, they help to assess the translational value of data generated by using human *in vitro* models.

Future work will focus on mechanistic investigations of liver effects triggered by immunomodulatory drugs and bi-specific antibodies. Here, mouse *in vivo* studies have demonstrated liver-specific immune-related side effects. Using our (innate) immune-competent mouse 3D liver spheroids, we are aiming to recapitulate and investigate these findings to better understand the mechanisms and assess the effect in terms of human relevance.

In conclusion, we anticipate that preclinical studies including 3D liver spheroids from rodent and non-rodent species will lead to better understanding of mechanism and pathways involved in drug-induced responses as well as more accurate predictions of drug-induced hepatotoxicity risks, ultimately optimizing the process of developing drugs with high efficacy and low safety concerns.

## Supporting information

S1 Fig Optimization of liver spheroids. Different amounts of hepatocytes (1000, 1250, 1500, 1750, or 2000 cells per spheroid) were seeded into ULA plates and monitored over 3 weeks in culture. Data are represented as average spheroid diameter ± standard deviation (n = 8 spheroids per condition).
(TIF)

S2 Fig Characterization of 3D liver spheroids. **a** Bright-field images show mouse-liver 3D-spheroid formation over 7 days. 1250 hepatocytes were seeded in plating medium containing 10% FBS (+FBS) into a round-bottom ultra-low-attachment plate on day 0 and centrifuged to allow sedimentation of cells at the center bottom of the well. From day 4 on, 50% of the cell culture medium was replaced daily with serum-free maintenance medium (-FBS). Scale bar = 100 μm **b** Nuclear staining of spheroid on day 21, scale bar = 50 μm.
(TIF)

S3 Fig Cyp3a activity of untreated liver spheroids over time. Cyp3a activity in both mono- and coculture spheroids were measured on day 4, 7, 11, 14, 18, 21, and 24 after plating. Data are expressed as average % of day 4 ± standard deviation (n = 8 spheroids/condition).
(TIF)

S4 Fig Cyp450 induction in liver spheroids. Cyp450 induction of spheroids treated for 3 days on day 7–10 (black bar) and day 14–17 (grey bar) with **a** 50 μM Pregnenolone 16α-carbonitrile (PCN) **b** 15 μM β-naphthoflavone (βNF) and **c** 100 μM Phenobarbital (Pb). Gene expression for CYP1Aa, CYP1A2, CYP2B6, and CYP3A11 mRNA was measured via qRT-PCR, data is represented as multiples of the change in the vehicle control ± standard deviation.
(TIF)

S5 Fig Cell viability of liver spheroids treated with Cyclosporine A. ATP-dependent viability of liver spheroids treated with 5, or 25 μM Cyclosporine A (CsA) for 48 hours. Data is represented as average ATP content per spheroid (pmol ATP/spheroid) ± standard deviation (n = 8 spheroids/condition).
(TIF)

S6 Fig Cell viability and LDH release of liver spheroids treated with Cyclophosphamide and Troglitazone. Spheroids were treated with 1.25, 2.5, or 10 μM Cyclophosphamide (CPZ) or Troglitazone for 24 h and analyzed for ATP content and LDH release. As positive control, 150 μM CPZ (+) was used. a Average ATP content of liver spheroids, data are represented in pmol ATP/spheroid ± standard deviation (n = 8 spheroids/condition) b Average LDH release of liver spheroids. Data are expressed as x-fold change compared to vehicle control (ctrl) ± standard deviation.
(TIF)
