# Supplementary figures and images for: Characterization of a long-term mouse primary liver 3D tissue model recapitulating innate-immune responses and drug-induced liver toxicity
Source: PLoS One. 2020 Jul 9;15(7):e0235745. doi: 10.1371/journal.pone.0235745 (PMC7347206; doi:10.1371/journal.pone.0235745)

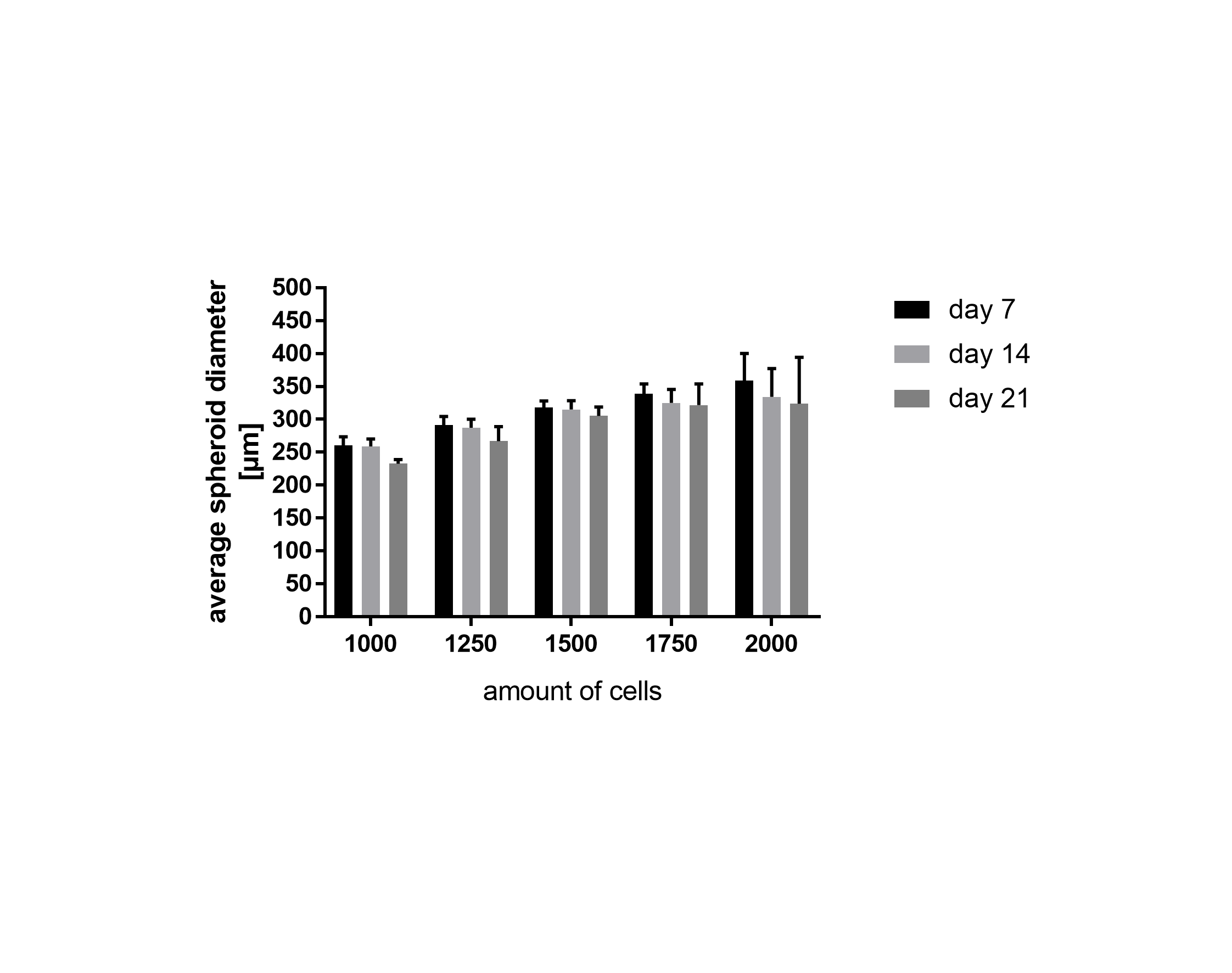

Supplement: S1 Fig — Different amounts of hepatocytes (1000, 1250, 1500, 1750, or 2000 cells per spheroid) were seeded into ULA plates and monitored over 3 weeks in culture. Data are represented as average spheroid diameter ± standard deviation (n = 8 spheroids per condition). (TIF) [file pone.0235745.s001.tif]

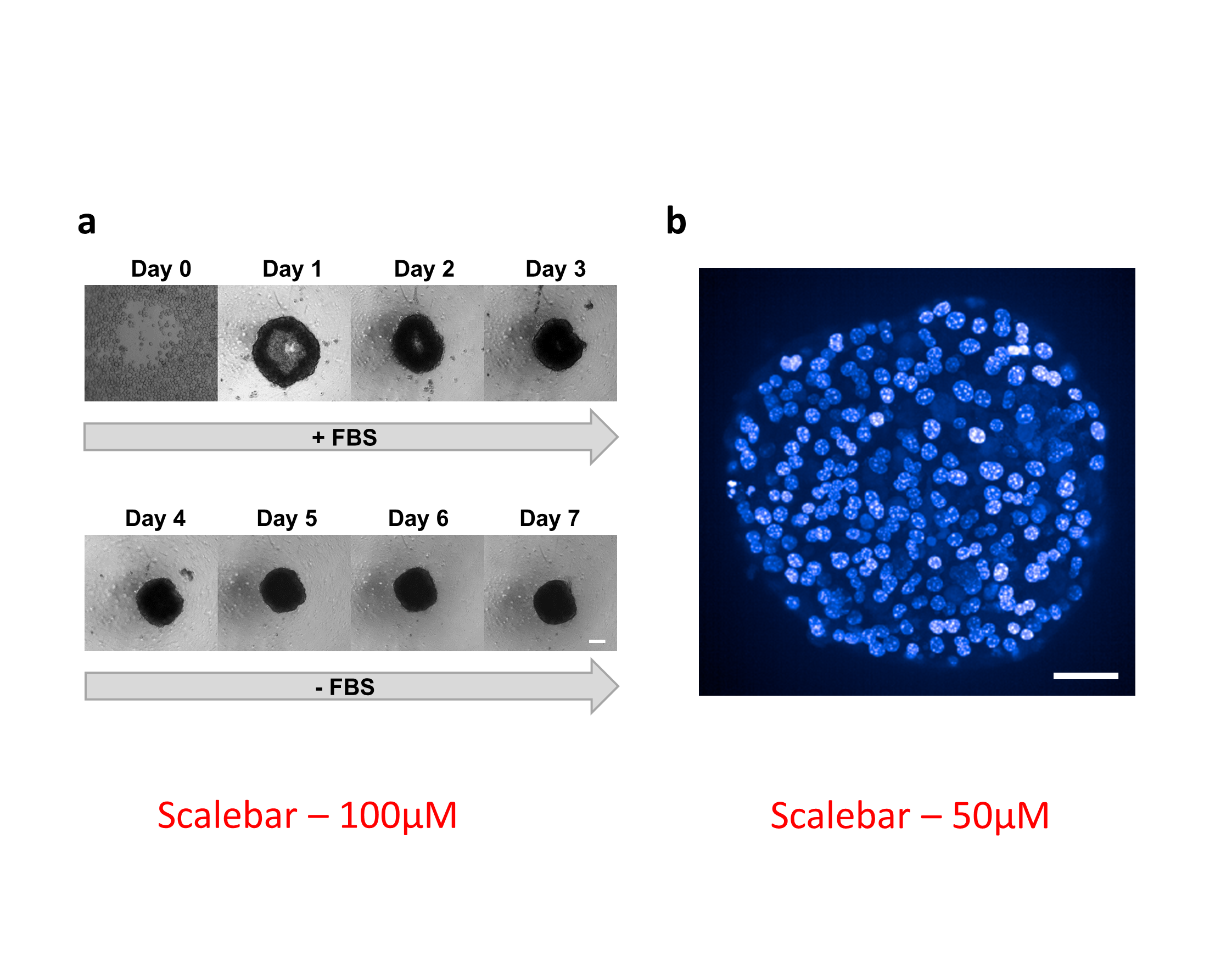

Supplement: S2 Fig — a Bright-field images show mouse-liver 3D-spheroid formation over 7 days. 1250 hepatocytes were seeded in plating medium containing 10% FBS (+FBS) into a round-bottom ultra-low-attachment plate on day 0 and centrifuged to allow sedimentation of cells at the center bottom of the well. From day 4 on, 50% of the cell culture medium was replaced daily with serum-free maintenance medium (-FBS). Scale bar = 100 μm b Nuclear staining of spheroid on day 21, scale bar = 50 μm. (TIF) [file pone.0235745.s002.tif]

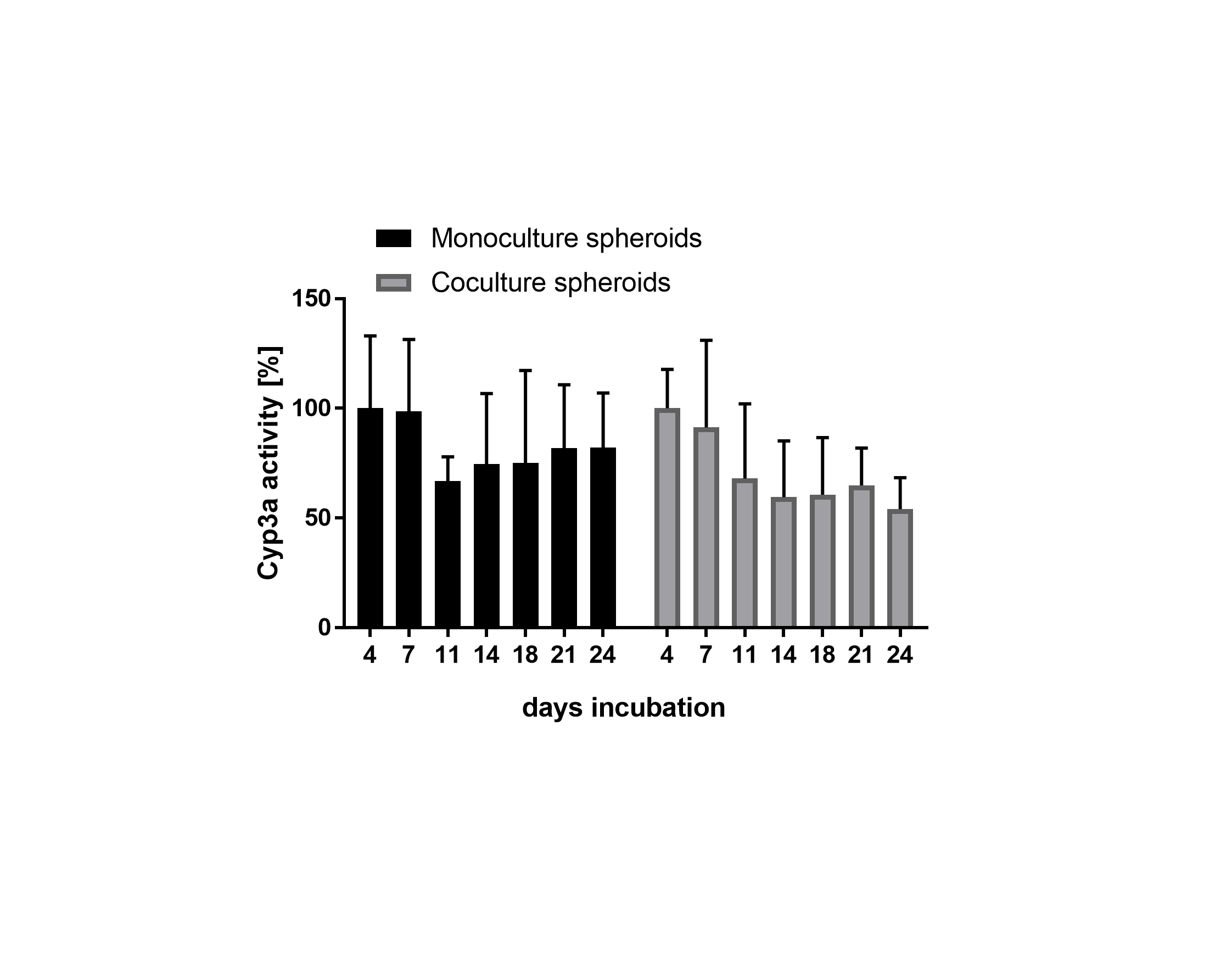

Supplement: S3 Fig — Cyp3a activity in both mono- and coculture spheroids were measured on day 4, 7, 11, 14, 18, 21, and 24 after plating. Data are expressed as average % of day 4 ± standard deviation (n = 8 spheroids/condition). (TIF) [file pone.0235745.s003.tif]

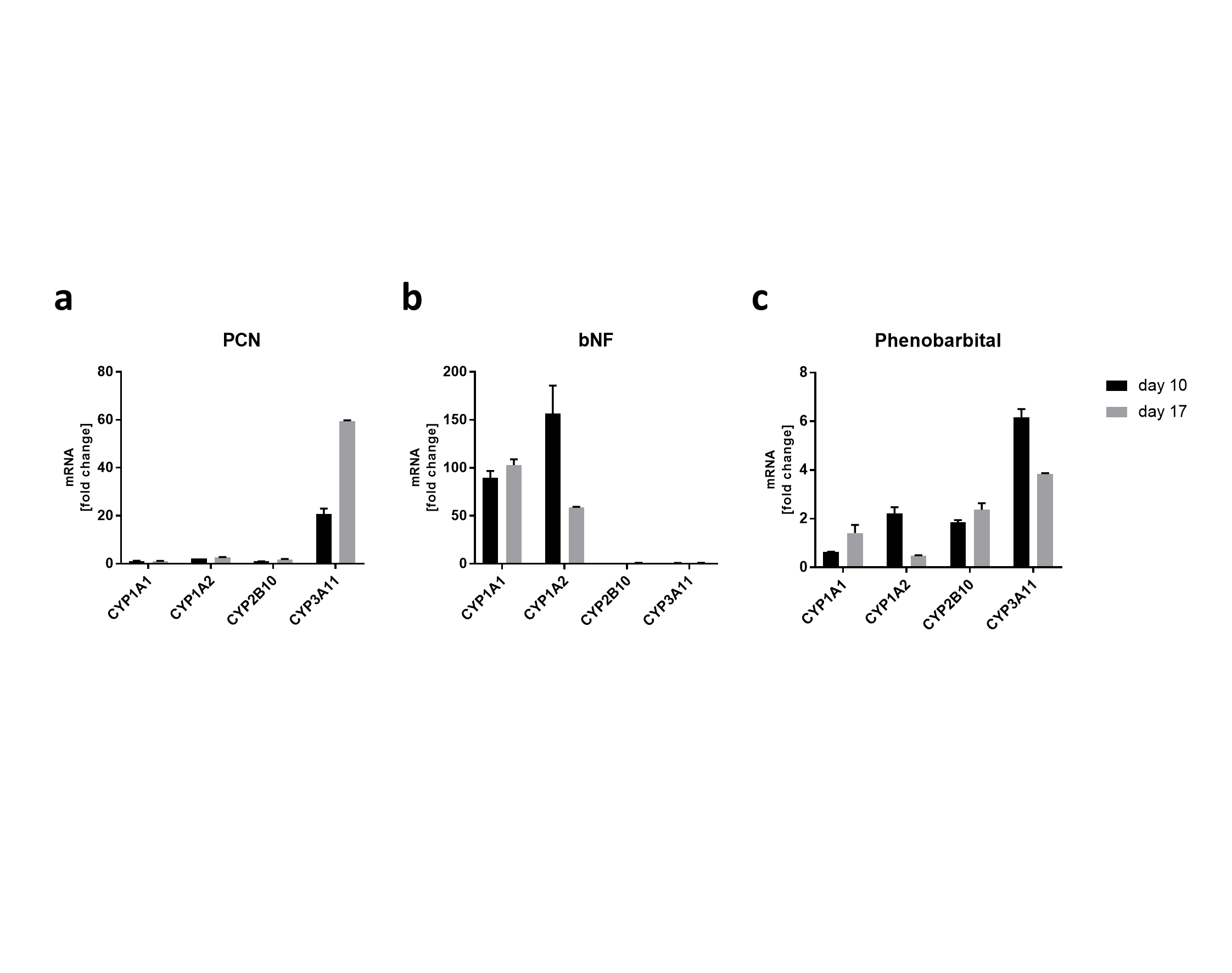

Supplement: S4 Fig — Cyp450 induction of spheroids treated for 3 days on day 7–10 (black bar) and day 14–17 (grey bar) with a 50 μM Pregnenolone 16α-carbonitrile (PCN) b 15 μM β-naphthoflavone (βNF) and c 100 μM Phenobarbital (Pb). Gene expression for CYP1Aa, CYP1A2, CYP2B6, and CYP3A11 mRNA was measured via qRT-PCR, data is represented as multiples of the change in the vehicle control ± standard deviation. (TIF) [file pone.0235745.s004.tif]

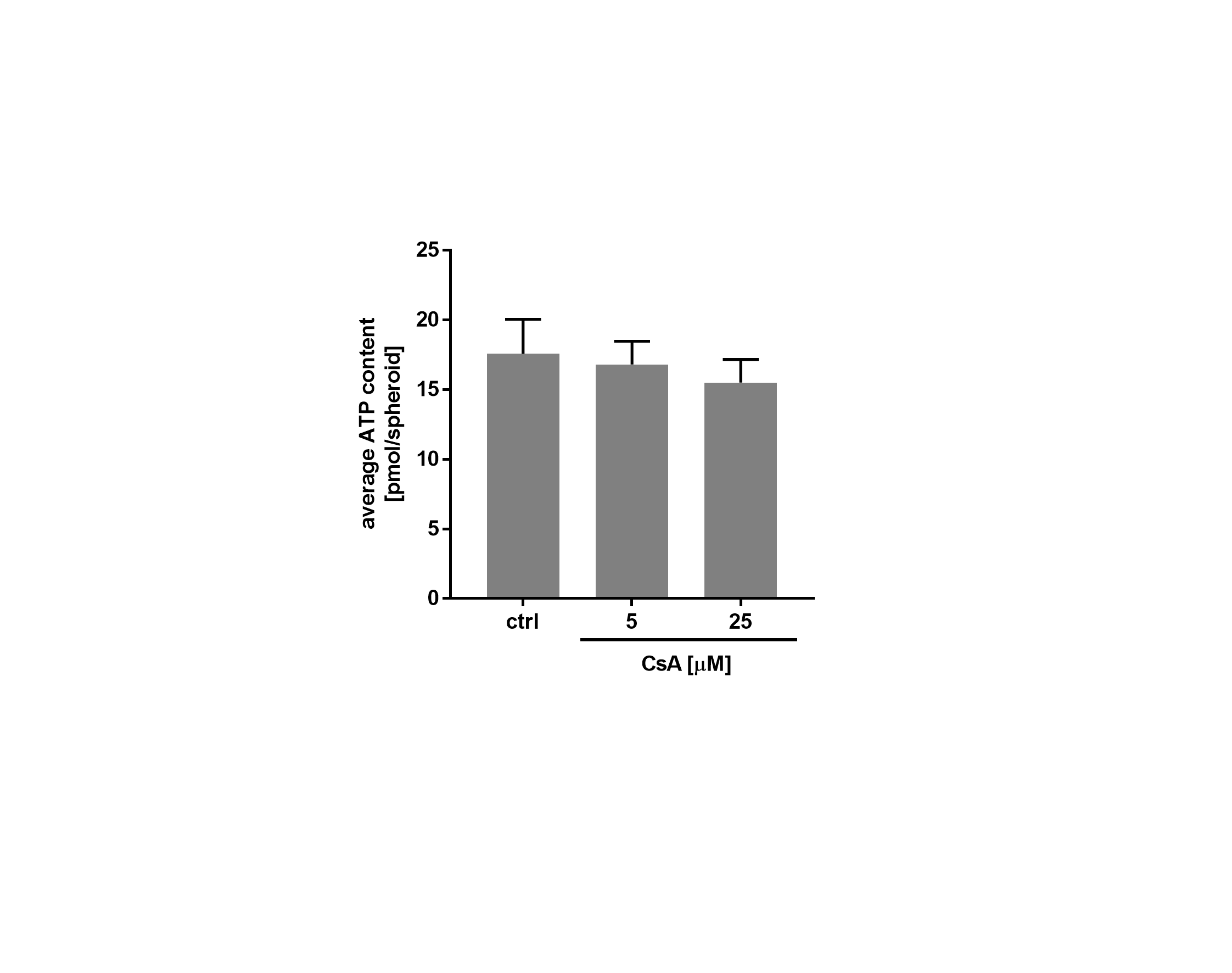

Supplement: S5 Fig — ATP-dependent viability of liver spheroids treated with 5, or 25 μM Cyclosporine A (CsA) for 48 hours. Data is represented as average ATP content per spheroid (pmol ATP/spheroid) ± standard deviation (n = 8 spheroids/condition). (TIF) [file pone.0235745.s005.tif]

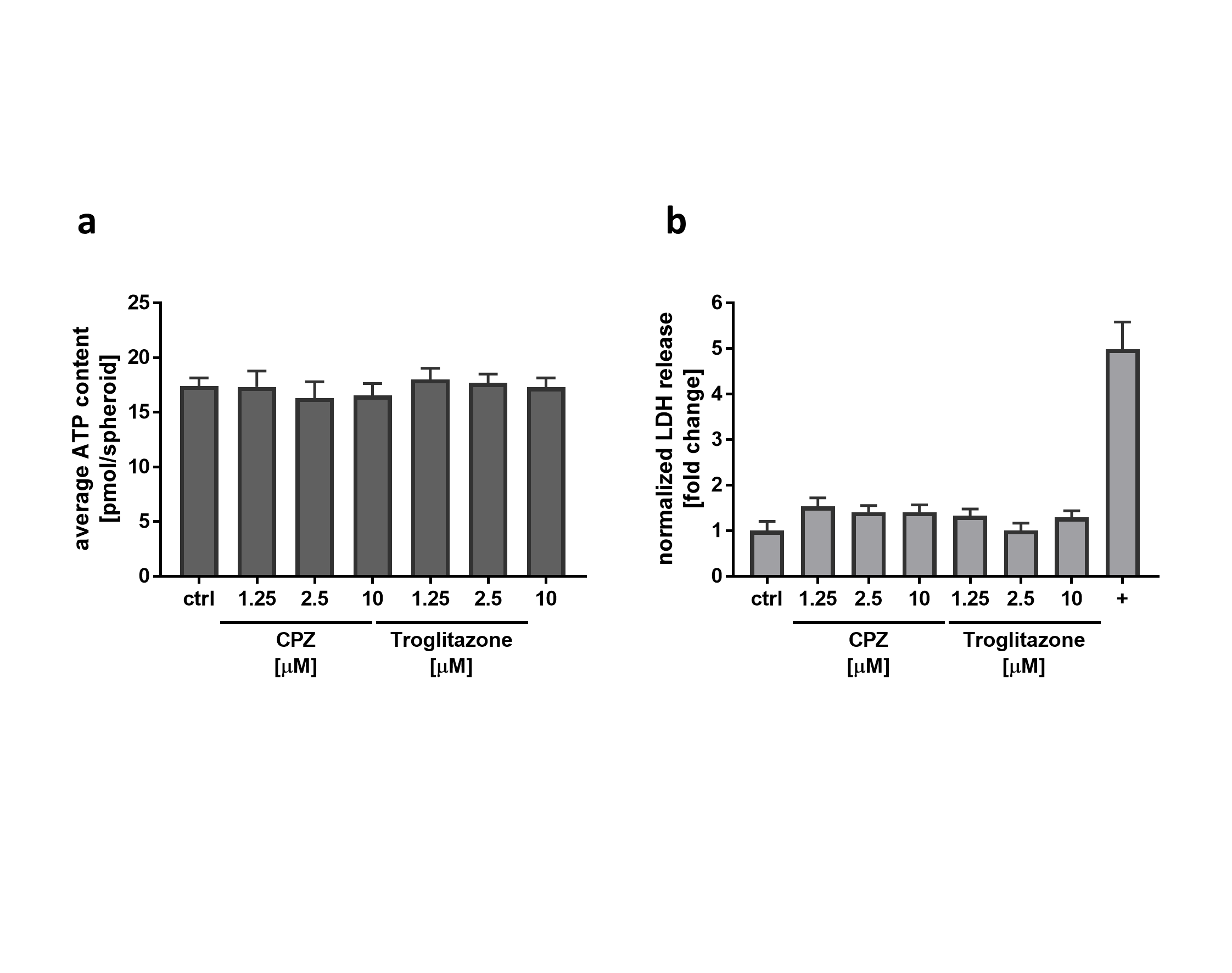

Supplement: S6 Fig — Spheroids were treated with 1.25, 2.5, or 10 μM Cyclophosphamide (CPZ) or Troglitazone for 24 h and analyzed for ATP content and LDH release. As positive control, 150 μM CPZ (+) was used. a Average ATP content of liver spheroids, data are represented in pmol ATP/spheroid ± standard deviation (n = 8 spheroids/condition) b Average LDH release of liver spheroids. Data are expressed as x-fold change compared to vehicle control (ctrl) ± standard deviation. (TIF) [file pone.0235745.s006.tif]
